# Biochemical adaptation in brain Acetylcholinesterase during acclimation to sub-lethal temperatures in the eurythermal fish *Tilapia mossambica*

**DOI:** 10.1038/s41598-019-56066-x

**Published:** 2019-12-24

**Authors:** Vijay Aswani, David Trabucco

**Affiliations:** 1 Department of Internal Medicine & Pediatrics, University at Buffalo, 1001 Main St, Buffalo, NY 14203 USA; 2 Jacobs School Of Medicine And Biomedical Sciences, University at Buffalo, 955 Main St, Buffalo, NY 14203 USA

**Keywords:** Hydrolases, Molecular fluctuations, Animal physiology, Ichthyology, Enzymes, Enzymes

## Abstract

*Tilapia mossambica* is a eurythermal tropical fish. We studied the effect of temperature on the kinetics of brain Acetylcholinesterase (AChE) during adaptation to sublethal temperatures by acclimating the fish to 37 °C, and controls to 25 °C. Electrophoresis showed the presence of two AChE bands that did not change in position or intensity with acclimation period or temperature. The apparent K_m_ was 0.23 ± 0.01 mM ATChI and remained relatively constant over the *in vitro* assay temperature range 10 °C to 40 °C. Biochemical characterization suggested that the enzyme is a ‘eurytolerant protein’ in its stability of kinetic and thermal properties over a wide temperature range. Thermal stability and arrhenius plots suggested that the AChE was made up of two forms that differed in their thermal properties.The two molecular forms of acetylcholinesterase were purified from the brain of *T. mossambica*. Molecular weight studies revealed that the two forms were size isomers: a monomer of 59 KDa and a tetramer of 244 KDa. They differed in their K_m_s, thermal stabilities and energies of activation. We suggest that biochemical adaptation to temperature in the brain acetylcholinerase system of the fish *Tilapia mossambica* is based on the aggregation-dissociation of these size isomers.

## Introduction

*Tilapia mossambica* currently known as *Oreochromis mossambicus* (Peters, 1852) is a subtropical fish whose ability to survive at high temperatures is reported to constitute an adaptation to its habitat of origin: the tropical river basins in Africa, where water temperatures upto to 35 °C have been recorded^1^. *Tilapia mossambica* withstands a wide temperature range^2^. Although its ability to adapt to sublethal temperatures was known^3^, few attempts have been made to look at the enzyme acetylcholinesterase (AChE) and how it changes with acclimmation temperature.

Lagerspetz^4^ reviewed understanding of the term ‘thermal acclimation’, comparing the definitions as laid out by Prosser, Precht and Bullock and pointed out that acclimation is common because it preserves genetic diversity and it allows transition from one steady state to another in open systems. On the basis of their thermal preference and their tolerance (determined experimentally and from their geographic distribution), *T. mossambica* was classified as a eurythermal species–one which tolerates a wide range of temperature^2^. We report here on the kinetic properties of AChE from the fish acclimated to a sub-lethal temperature to determine whether any adaptive mechanism was operative. The enzyme AChE was selected because previous work on other species has shown that during thermal acclimation, adaptive changes in the kinetic properties of AChE are usually observed^5^. On the basis of assays of brain acetylcholinesterase, Pradhan^6^ postulated the existence of isozymes, but did not demonstrate them.

## Results and Discussion

### Acclimmation

**Figure 1:**
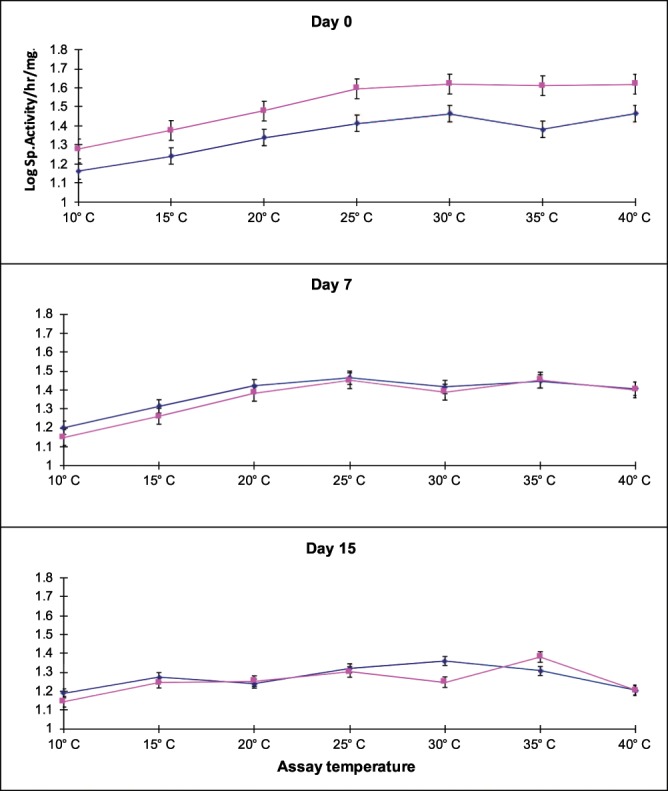
Change in brain AChE activity at different assay temperatures during acclimation. Shown is activity at the start (Day 0), mid-way (Day 7) and at the end (Day 15) of the acclimation period (25 °C: blue line and 37 °C: pink line).

Figure 1 shows the change in the V_max_ of acetylcholinesterase during the 15-day acclimation period. There was a reduction in rate function (V_max_) noted by day 7 of acclimation to the higher temperature, compared to the baseline of the fish at 25 °C (control). The figure shows some difference in AChE activity on day 0 between the fish in the control and acclimation tanks. The fish were separated from the stock tank into the control and acclimation tanks on that day. It is possible that the difference between the two tanks on day 0 represent some induced in the fish from the transfer or the difference between them was the difference between two samples of a population. This suggested an adaptive compensatory response to the higher temperature. It appeared from these data that two weeks was a sufficient period for adaptation to occur in the nervous system of *Tilapia*. Prosser reported that adaptation in the case of nervous system occurs faster than in other systems – in a few days, while metabolic adaptations often take as long as a month^7^.

### Electrophoresis

**Figure 2:**
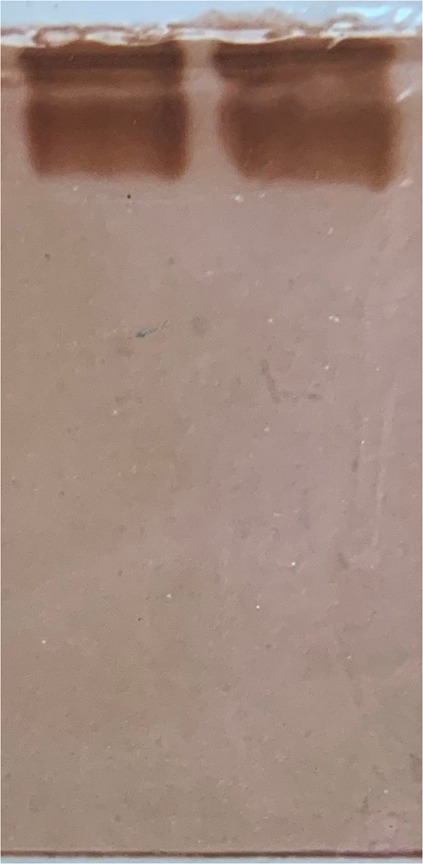
PAGE of crude brain AChE showing 2 bands. Lanes from left to right: AChE from fish at 25 °C, AChE from fish at 37 °C.

Since earlier workers had reported that such compensation is often the result of different isozymes acting at different temperature ranges^5^, attempts were made to localize the AChE species extracted from fish acclimated to 37 °C, compared to the control. The AChE band pattern of the 37 °C acclimated fish was no different than the control (Fig. 2). This pattern obtained was found to be very similar to that obtained from the brain and nervous tissue of other species^8–11^ in which 2 bands were observed with no variation between different temperature-acclimated organisms. It may be argued that if the slower moving band represents the form of AChE better suited to function at 37 °C and the faster moving band represents the form better suited to function at 25 °C, then electrophoresis gels of fish acclimated to these temperatures should show relative increases of these forms at the respective temperatures. This was not seen. A possible explanation for this could be that the electrophoresis was run at 4 °C and that the size isomers loaded onto the gel at 4 °C might have equilibrated in a fixed ratio suitale to the temperature. It may be technically difficult to show the dominant form of AChE at the temperature of acclimation.

The phenomenon of different isozyme forms in populations of a species acclimated to different temperatures may be restricted largely to species like salmonids, e.g. the rainbow trout, where gene duplication has generated a large enzyme repertoire. Therefore the finding of no change in isozyme pattern with acclimation was not surprising and likely the most common reponse expected in a diploid species. Merchant *et al*.^12^ built upon the work reported in this paper and its associated PhD thesis^13^. They studied I brain AChE from fish isolated in summer (mean water temperature 30 °C) and winter (mean water temperature 18 °C). Their work concluded that the enzyme AChE is a eurythermal enzyme protein with maximum substrate affinity between 25 °C and 30 °C.

### Change in kinetic parameters with temperature

Kinetic data obtained from apparent K_m_ versus *in vitro* temperature plots seemed to indicate little change in K_m_ over the range 10 °C to 40 °C (Table 1).

**Table 1:** K_m_ (mM of ATChI) values of brain AChE versus assay temperature at different days of acclimation.

Assay	25 °C			37 °C		
temp	0	7	15	0	7	15
10 °C	0.2 ± 0.007	0.15 ± 0.005	0.18 ± 0.006	0.2 ± 0.007	0.14 ± 0.005	0.14 ± 0.005
15 °C	0.16 ± 0.006	0.29 ± 0.010	0.18 ± 0.006	0.2 ± 0.007	0.26 ± 0.009	0.25 ± 0.009
20 °C	0.19 ± 0.007	0.21 ± 0.007	0.17 ± 0.006	0.22 ± 0.008	0.27 ± 0.009	0.23 ± 0.008
25 °C	0.22 ± 0.008	0.21 ± 0.007	0.28 ± 0.010	0.27 ± 0.009	0.19 ± 0.007	0.23 ± 0.008
30 °C	0.22 ± 0.008	0.27 ± 0.009	0.21 ± 0.007	0.2 ± 0.007	0.24 ± 0.008	0.23 ± 0.008
35 °C	0.29 ± 0.010	0.28 ± 0.010	0.18 ± 0.006	0.39 ± 0.014	0.29 ± 0.010	0.26 ± 0.009
40 °C	0.26 ± 0.009	0.28 ± 0.010	0.21 ± 0.007	0.29 ± 0.010	0.32 ± 0.011	0.28 ± 0.010

This would be consistent with the fact that *Tilapia mossambica* is a known eurythermal fish. The data suggested that the AChE in the brain of *Tilapia mossambica* is a eurythermal or eurytolerant protein similar to the AChE of the mullet fish (*Mugil cephalus*) reported by Somero^14^. AChE from *T. mossambica* showed a similar apparent K_m_ to that of *Mugil cephalus*: a similar variation in K_m_, ranging from 0.12 to 0.3 mM ATChI over the *in vitro* temperature range 10 °C to 40 °C.

### Thermal stability

**Figure 3:**
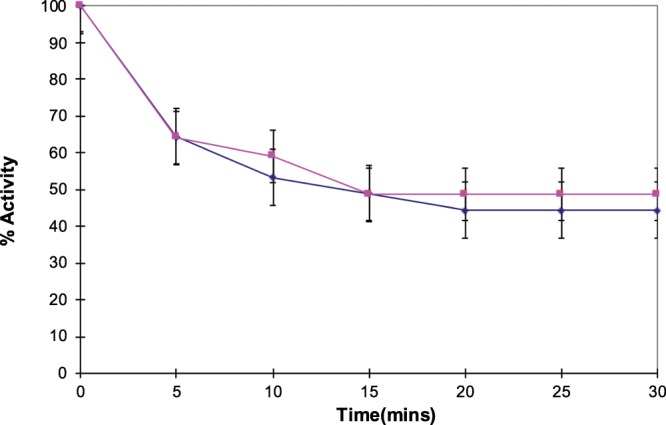
Thermal stability of crude brain AChE incubated at 45 °C for 30 minutes, showing an initial rapid and subsequent slower fall in residual activity over time. Time 0 activity – taken as 100% was 0.0142 Units of AChE per ml of brain extract.

The thermal stability profiles of the membrane-bound AChE suggested the presence of a thermolabile fraction (the initial rapid decrease in activity with time) and a relatively thermostable fraction (the slower decrease in activity) (Fig. 3).

### Arrhenius plots

**Figure 4:**
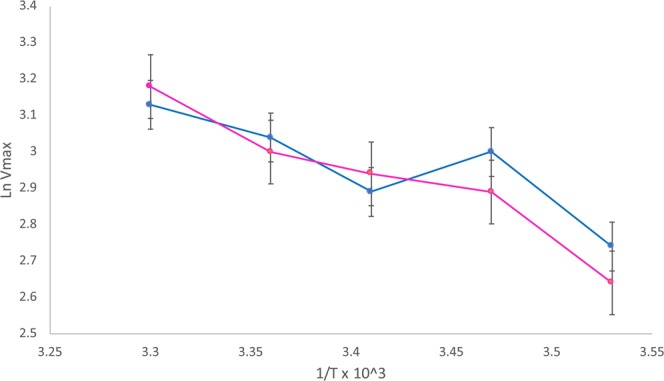
Arrhenius plot of brain AChE (25 °C: blue line and 37 °C: red line).

The plot of V_max_ versus 1/T of membrane-bound AChE showed a break in the curve (Fig. 4). This could be suggestive of a transition between two different conformations or molecular forms of the AChE^15,16^. Evidence has been presented for similar temperature-dependent transitions between multiple forms of serum cholinesterase and AChE from erythrocytes by Main^17^.

These data collectively suggested that the membrane-bound AChE is eurytolerant protein as defined by Somero^14^: ‘a single form of a protein which is capable of maintaining its structural and functional characteristics, within an acceptable range over the entire spectrum of environmental conditions faced by the organism (enzyme).’

Data for other enzymes and fishes, such as the eurythermal *Gillichthys mirabilis*^18^ and the pyruvate kinase enzymes^19,20^ showed the eurytolerant strategy of adaptation to be more favoured and optimal at least for fishes (unlike plants, in which polyploidy would permit the isozyme selection strategy of adaptation^21^).

### Purification

The AChE species were isolated from a pooled sample of 70 adult fish brains taken from fish held at 25 °C. The extraction procedure yielded 66% of the total activity in the detergent-soluble phase (Table 2).

**Table 2:** Distribution of AChE activity in the different stages of solubilization of the enzyme from the brain of *T. mossambica*.

Stage	Volume (mls)	Total AchE Units (µmol/min)	Total protein (mgs)	Sp. Activity Units/mg	Yield %
Homogenate	71.30	147.05	429.50	0.3424	100.00
‘Soluble’ Centrifugate	66.00	38.04	151.80	0.2506	25.87
Membrane-bound centrifugate	70.00	97.65	268.90	0.3631	66.41
Pellet	25.00	12.89	79.98	0.1612	9.45

Analytical non-denaturing PAGE of the membrane-bound AChE extract showed the prescence of a broad, faster moving band (Band I) and a sharp, slower band (Band II) (Fig. 2).

Preliminary attempts to purify the two molecular forms of AChE by gel filtration or ion-exchange chromatography proved unsuccessful. The single peaks observed in both these profiles, in spite of two bands observed on PAGE could be explained by the tendency of AChE to aggregate^11,22,23^.

**Figure 5:**
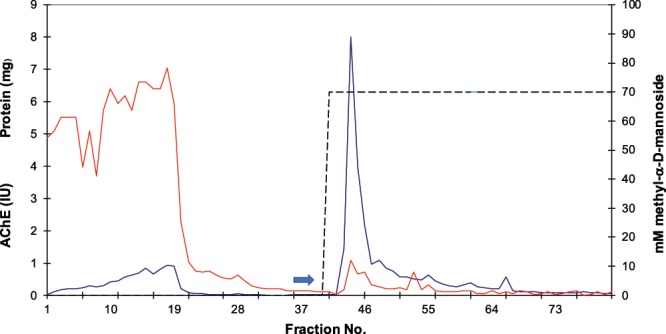
Con A Sepharose 4B lectin chromatography of 50% ammonium sulphate precipitate re-dissolved. Arrow indicates start of elution using 70 mM methyl-α-D-mannoside in starting buffer [solid blue line, AChE activity Units/ml; solid red line, protein mg/ml]; dashed black line eluting buffer.

**Figure 6:**
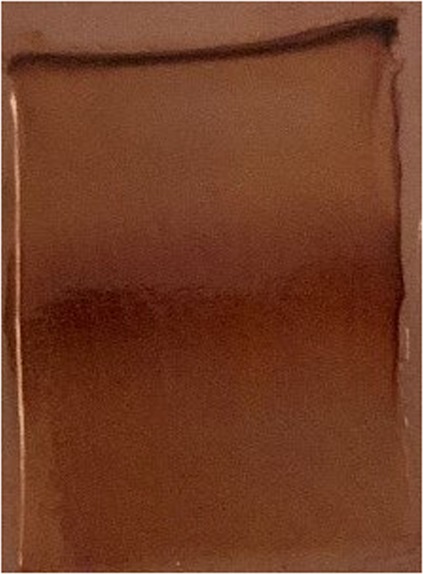
Longitudinal section of the preparative PAGE gel showing the position of the two molecular forms of AChE. The corresponding positions in the unstained gel were cut out and purified by electroelution.

The final purification procedure involved Con A affinity chromatography (Fig. 5) and preparative PAGE, followed by electroelution (Fig. 6). The procedure yielded Band I with a fold purification of 2.63 and Band II with a fold-purification of 18.43, with yields of 0.37% and 2.61%, respectively.

The over- all yield of the purification process was 3% of the total brain AChE, and 4.5% of the total membrane-bound AChE extracted (Table 3). The low fold purification figures were most likely because of the high degree of thermal denaturation during the preparative PAGE step. However, the process successfully resolved Bands I and II AChE into apparently homogeneity fractions.

**Table 3:** Purification of two molecular forms of AChE from the brain of fish *T. mossambica*.

Stage	Volume (mls)	Total AChE (µmol/min)	Total protein (mgs)	Sp. Activity Units/mg	Fold Purification	Yield %
Homogenate	71.30	147.05	429.50	0.3424	1.00	100.00
Triton-solubilzed fraction	70.00	97.65	268.90	0.3631	1.06	66.41
50% satd. (NH_4_)_2_SO_4_ fraction	64.00	69.88	166.54	0.4196	1.22	47.52
Con A Sepharose eluate	5.20	10.60	2.14	4.9890	14.57	7.21
Preparative PAGE:						
Band I	44.00	0.55	0.61	0.9006	2.63	0.37
Band II	92.00	3.84	0.61	6.3100	18.43	2.61
					Total Yield:	2.98

The molecular weight of the slow-moving AChE, Band I was found to be 244 KDa while that of the faster-moving Band II was found to be 59 KDa by non-denaturing PAGE using a Ferguson plot method extrapolating from molecular weight markers in different percentage cross-liked PAGE. The molecular weights seem to correspond closely to those obtained for the monomer and tetramer of G_4_ AChE, particularly the ‘11 S’ form^24–27^.

The data from native and denaturing PAGE therefore suggested that Band I was a tetrameric form of Band II, both having identical sub-units. This was confirmed by SDS-PAGE, which yielded a single band.

### Enzyme kinetics

The two purified AChE bands showed very different kinetic properties. In both Band I and Band II, the K_m_ was found to increase with temperature (Table 4). This would suggest a decreased affinity of the enzyme for the substrate with increasing temperature. This could compensate for the increased velocity of the reaction due to increased temperature. The K_m_ of Band I was about 10 times greater than of Band II.

**Table 4:** Km (mM ATChI) and Vmax (µmol/min/g protein) of Bands I and II AChE at different assay temperatures.

Assay	Band I		Band II	
temp	Km	Vmax	Km	Vmax
10 °C	0.4 ± 0.014	0.0073 ± 0.000	0.21 ± 0.007	0.0323 ± 0.001
15 °C	2.0 ± 0.070	0.0255 ± 0.001	0.14 ± 0.005	0.042 ± 0.001
20 °C	2.4 ± 0.084	0.0508 ± 0.002	0.21 ± 0.007	0.0584 ± 0.002
25 °C	5.38 ± 0.188	0.1691 ± 0.006	0.17 ± 0.006	0.0721 ± 0.003
30 °C	2.5 ± 0.088	0.1568 ± 0.005	0.27 ± 0.009	0.1097 ± 0.004
35 °C	8.42 ± 0.295	0.6154 ± 0.022	0.36 ± 0.013	0.1466 ± 0.005
40 °C	4.54 ± 0.159	0.5752 ± 0.020	0.52 ± 0.018	0.208 ± 0.007

The V_max_ versus temperature plots suggested some transition in catalytic function from Band II to Band I with increase in temperature: below 20 °C, the V_max_ of Band II was greater than that of Band I. Above 20 °C, the V_max_ of Band I was greater than that of Band II. The point of inflection seemed to be at around 20 °C.

### Thermal stability

**Figure 7:**
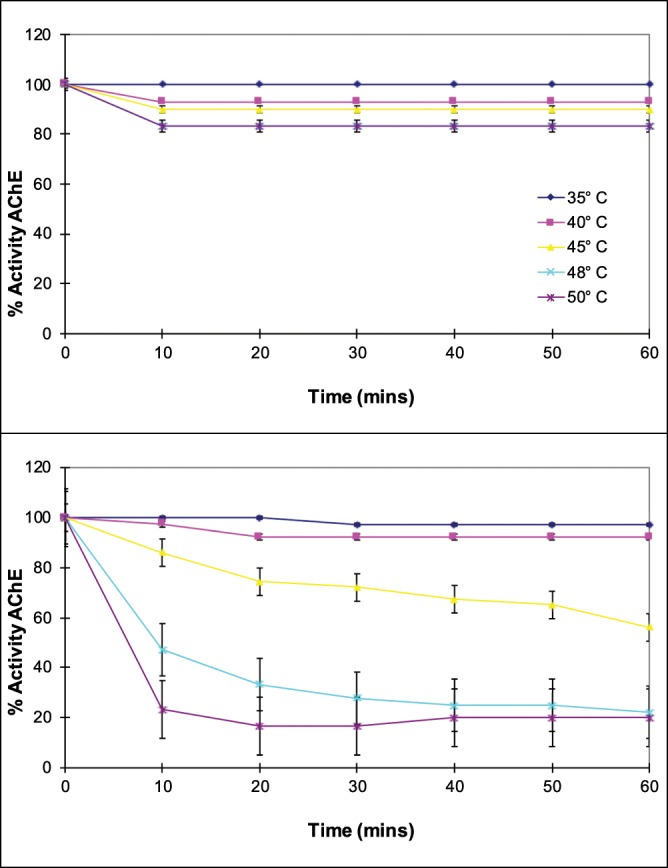
Thermal stability of Bands I (top panel) and II (bottom panel) (blue: 35 °C, pink: 40 °C, yellow: 45 °C, light blue: 48 °C, purple: 50 °C).

The thermal stability profiles of the two Bands confirmed results obtained earlier where a two-sloped thermal stability profile was obtained with a crude AChE extract. This suggested that the crude extract was composed of a thermolabile and a relatively thermostable fraction. Band I was more stable than Band II (Fig. 7). When exposed to 50 °C for 1 hour, only 20% of the activity of Band I was lost as compared to 80% activity lost in the case of Band II.

Since Band I was found to be a tetramer of Band II, the greater thermal stability could be explained in that some energy would go into breaking the inter-molecular bonds before denaturation of the individual monomers could occur.

Consistent with the picture emerging from the kinetic studies that Band I probably contributed more to catalytic activity at a higher temperature is the observation that it was significantly more stable at higher temperatures. Thus, the brain AChE of *T. mossambica* could undergo a transition from monomer to tetramer form by reversible association with increase in temperature. This simple physical change would render the AChE more stable and its kinetic parameters better suited for function at the higher temperature.

### Arrhenius plots

**Figure 8:**
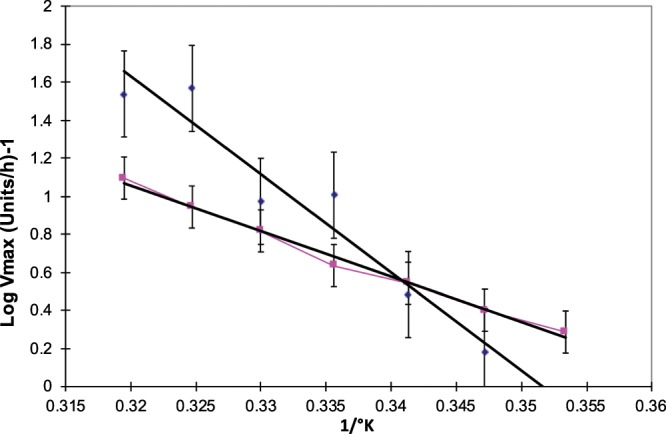
Arrhenius plots for AChE Bands I (in blue) and II (in pink).

The Arrhenius plots of purified Bands I & II were both linear (r = −0.98 for Band I and r = −0.99 for Band II) (Fig. 8).

Arrhenius plots of crude membrane-bound AChE yielded biphasic curves (Fig. 3). The fact that the Arrhenius plots of both Band I & Band II were linear suggested that the biphasic curves obtained with the crude extract were due to the presence of two molecular forms of AChE with different thermal properties.

The E_a_ for Bands I & II were found to be 27.27 Kcals.mole^−1^ and 11.39 Kcals.mole^−1^ of substrate, respectively. The higher energy of activation for Band I would be consistent with its proposed function at higher temperatures. By decreasing the catalytic efficiency of the enzyme, the rate of reaction can be maintained constant in spite of increased temperature. This strategy is seen in evolution of homologous proteins^18^ for e.g. E_a_ for homeothermic organisms is often higher than that for the corresponding enzyme/protein of ectothermic organisms living at lower temperatures.

Observing the break point in Arrhenius plots of the crude enzyme, it was felt possible that a reversible conversion of monomer to tetramer might be feasible by changes in the environment of the molecule. Further, a break in Arrhenius curves of crude membrane-bound AChE has been interpreted to be due to a transition between two different conformations or molecular forms of the AchE^15,16^.

Vidal *et al*.^28^ studied AChE from rat brain and found that K_*m*_ for detergent soluble enzyme was higher a buffer-soluble preparation. They also noted a break in the Arrhenius plot at 23 °C for the detergent soluble enzyme. They found that the lowest energy of activation was 13 kJ mol-1 at the physiological temperature of 37 °C. Using a pH gradient PAGE, they obtained bands corresponding to molecular weights of 70 to 450 KDa. They report that lowest molecular weight band of 70 KDa is probably the monomer corresponding to the globular G1 forms reported in several species and the 270 KDa is likely the tetramer similar to the AChE from pig.

Adamson *et al*.^29^ demonstrated a monomeric form of brain acetylcholinesterase (molecular weight: 77 KDa) in mouse brain that subsequently formed dimers and tetramers. Nemczok^30^ showed that the aggregation and dissociation of molecular forms of AChE may be altered by chemical or biochemical stimuli, in their case, the action of an insecticide. It has been recognized for some time that polymeric proteins may show association and dissociation of their subunits and that this dynamic conversion may depend upon physical changes in the environment. Such properties of polymeric enzymes have been exploited by organisms in adaptation to temperature^31^. Argos *et al*.^32^ suggested that changes in hydrophicity might change thermal stability in enzymes such as lactate dehydrogenase and glyceraldehyde-3-phosphate dehydronase. Hochachka^33^ studied the effect of temperature and pressure adaptation on the binding site of acetylcholisterase and showed that the hydrophic contribution to binding at the anionic site was strongly disrupted at low temperatures and high pressures.

Baldwin^34^ studied enzyme kinetics of brain acetylcholinesterase from fish isolated from different ambient water temperature settings: *Trematomus borchgrevinski* from the Antartic, *Mugil cephalus* and *Parupeneus chryserydros* from Hawaii and several tuna species from Hawaii. He could not find a correlation with changes in K_*m*_ associated with changes in reaction rates over the temperature ranges studied. He postulated that a possible adaptive function fo the K_*m*_-temperature relationship may be an adaptive mechanism to maintain satisfactory conformational flexibility characteristics of the membrane throughout different temperatures ranges, since AChE is a phospholipid bound enzyme of neural membranes.

The study of various properties of the two brain AChE Bands isolated suggested their suitability for function at different temperature ranges. The various parameters explored consistently fitted in with this view. Hochachka and Somero developed an integrated model of biochemical adaptation that incorporated enzyme-substrate affinity, enzyme structural stability, activation energy and V_*max*_ into a coherent whole^35,36^.

The conversion of a monomer to a tetramer form of AChE, resulting in a renewed suitability of the enzyme to its new temperature environment would, in effect, give the organism the better of two ‘functional isoenzymes’ at the expense of a single gene*. T. mossambica* is a eurythermal organism and the data presented here suggested that molecular adaptation to transient changes in the environment may not require the investment of permanent genetic changes.

In his remarkable review of adaptation of enzymes to temperature and a homage to Peter Hochachka, a doyen in the field, Somero^37^ described three “strategies” of adaptation: changes in amino acid structure leading to adaptive variation in kinetic properties and structural stability of enzymes, shifts in concentrations of proteins through gene expression and protein turnover and finally changes in the milieu in which proteins function, modulating their activity in response to physiological needs. It is this third that is exemplified by the association and dissociation of brain AChE in adaptations to temperature to maintain hemostatic activity.

In recent years, it has been shown that acetylcholinesterase in the brains of mammals exist as a AChE_T_ variant^38^. Perrier *et al*.^39^ showed that this catalytic monomer is organized as a tetramer by anchoring it to the neuronal cell membrane via a proline-rich membrane (PRIMA). We performed a tblastn search using BLAST and filtering for *Oreochromis* and *Tilapia* and found a match with a *Oreochromis niloticus* mRNA (Nucleotide sequence XM_005477000.4 found at https://www.ncbi.nlm.nih.gov/search/all/?term = XM_005477000.4) that showed the Proline-rich domain found in the PRIMAs of mammals (humans and mice). While we do not have sequence from *T. mossambica* to verify this, it is highly likely that PRIMA is involved in anchoring the AChE tetramer form to the cell membranes of neurons. Xie *et al*.^40^ further showed that extractions at low concentrations of Triton X-100 (they used 0.05%) isolated a AChE G_4_ form (T variant) anchored by PRIMA into cholesterol-sphingolipid-rich membrane microdomains called membrane rafts, that are involved in synaptic signaling and plasticity. Bon and Massoulie^41^ demonstrated that under certain cellular conditions, varius oligomeric forms of acetylcholinesterase exist.

There is plenty of room for interesting investigations to unite the work of Hochachka and Somero in thermal adaptation strategies with the new studies on the amino acid sequence^42^, protein interactions and placement in the cell membrane of neurons of AChE^43^. For example, the effect of temperature on the solubility of membrane rafts, or on the hydrophobic interactions of PRIMA with AChE and their effects on enzyme kinetics remain to be explored. Our work here suggests that physico-chemical factors such as temperature might affect association-dissociation of AChE oligomers. However, the effect of temperature may not be so direct. Rather, it may be mediated through changes in the fluidity of the cell membrane, or on the effect of hydrophic interactions between the AChE subunit and its anchors. Furthermore, the kinetics of the different oligomers may need to be evaluated *in situ*, if possible.

## Methods

### Materials

Acetylthiocholine iodide, 5,5′-dithiobis(2- nitrobenzoic acid), Ethopropazine hydrochloride and tris were purchased from Sigma Chemical Co., St.Louis, Missouri, USA. Acrylamide and bis-acrylamide were obtained from Pfizer, Germany, TEMED from Koch-Light, England. Sephadex G25, DEAE Sephadex A_50_, Sephadex G200 and Concavalin A Sepharose 4B were obtained from Pharmacia fine Chemicals, Sweden. All other chemicals were from BDH AnalaR or E. Merck pro Analyst.

### Acclimation of fish

Adult *Tilapia mossamabica* of both sexes weighing 150 to 200 g (approximately 4 inches in length), were captured from the Vasundi freshwater lake in the district of Thane (Maharashtra state, India). After stabilization in the lab in a holding tank at 25 °C, they were tranferred to two identical seasoned freshwater tanks (115 × 40 × 40 cms; 75 L capacity), one tank serving as the sub-lethal temperature tank, the other as control. In each replicate of the experiment, each tank contained 24 fish. The lethal temperature of the *Tilapia* population used for the experimental tank was determined by preliminary experiments and found to be 39 °C. A sub-lethal temperature of 37 °C was selected for the acclimation temperature (mortality <5%).

The temperature in the sub-lethal temperature tank was raised from room temperature (25 °C) by 1.5 °C a day until the acclimmation temperature of 37 °C was reached. 7 fish each, from the experimental and control tanks were removed and sacrificed on days 0, 7 and 15 of the acclimation. This experiment was replicated thrice with similar results.

#### Approval

All experimental protocols were approved by the Mumbai University Research Committee and the methods were carried out in accordance with their relevant guidelines and regulations.

### Preparation of AChE

The extraction was carried out at 4 °C. Individual brains (weighing about 100 mgs) were homogenized in 0.32 M sucrose, 50 mM phosphate buffer, pH 7.5, 1 mM EDTA in a Potter-Elvejham homogenizer to prepare a 2% (w/v) homogenate. The extract was then centrifuged at 105,000 g for 1 h at 4 °C in a Centrikon T-1065 ultracentrifuge (Kontron Instruments, USA). The supernatant was the source of the soluble AChE. The pellet was re-homogenized as described above in the same buffer containing 0.1% Triton X-100 and centrifuged as before. This supernatent contained the membrane-bound AChE. The extract were dialyzed against the above buffer without sucrose and EDTA and used for determination of AChE activity and protein. For the purpose of electrophoresis and the determination of Michaelis-Menten parameters, the extracts of 5 to 7 individual brains were pooled. The extracts were stored at 0° to 4 °C and kinetics done on the same day as extraction to minimize proteolytic degradation.

### Assay of AChE activity

AChE activity was measured at 25 °C unless otherwise stated, by the method of Ellman *et al*.^44^. The enzyme activity was expressed in IU (μmol substrate hydrolyzed/min). Unless otherwise stated, the assay solution contained 1 mM acetylthiocholine iodide and 0.91 mM 5,5′-dithiobis(2- nitrobenzoic acid) in 100 mM phosphate buffer, pH 8. Ethopropazine hydrochloride was added in the reaction mixture in the final concentration of 10^−4^ M to inhibit pseudocholinesterases^45^.

Protein was determined according to the methods of Lowry *et al*.^46^, and by Peterson^47^, where interfering substances were present.

### Electrophoresis

AChE preparations were examined by polyacrylamide electrophoresis PAGE^48^, using the buffer system of Dewald *et al*.^49^, modified by increasing the Triton x-100 concentration to 1.0% (v/v) throughout. PAGE was carried out at 4 °C using a 3% stacking gel and 7% seperating gel. AChE bands were localized by the method of Karnovsky & Roots^50^. Protein staining was carried out using the silver staining method of Blum *et al*.^51^. Glycoprotein staining was carried out by the method of Zacharius *et al*.^52^.

### Enzyme kinetics

The Michaelis-Menten kinetic parameters were determined *in vitro* at temperatures from 10 °C to 40 °C at 5 °C intervals, in the substrate concentration range, 0 mM to 1.2 mM ATChI. The constants were determined by the direct linear plot^53^ using a computer program developed by Ireland & Long^54^.

## Purification of AChE

### Solubilization of Membrane-bound AChE

All operations were done at 0–4 °C. About 70 brains were dissected out of adult *Tilapia mossambica* specimens and used as the source of AChE. A 10 g % (weight of wet brain/volume of buffer) extract of fish brain was prepared by homogenizing the tissue with extraction buffer (0.32 M sucrose, 50 mM phosphate buffer, pH 7.5, 2 mM EDTA) in a Potter-Elvejham homogenizer. The homogenate was centrifuged at 18,000 g for 30 min. The pellet was re-homogenized with 10 volumes of the above buffer containing 1% v/v Triton X-100, then centrifuged as desribed above.

The supernatent thus obtained was carried over to 50 saturation with (NH_4_)_2_SO_4_. The precipitate was redissolved in a minimum volume of 50 mM phosphate buffer, pH 7.5, 0.5 M NaCl, 1% (v\v) Triton X-100 and desalted on a Sephadex G-25 column.

### Con A Sepharose 4B affinity chromatography

The desalted membrane- bound AChE extract (69.88 IU and 166.54 mgs protein in 64 ml) was applied to a column (1 cm × 1.8 cm) containing 1.4 ml of Con A Sepharose 4B. The column was washed with 80 ml of starting buffer and then eluted with starting buffer containing 70 mM methyl-α-D- mannoside.

#### Preparative PAGE

Preparative gel electrophoresis of the AChE peak was carried out in a unit fabricated in our laboratory. The gel tube was 2.2 cms x 12 cms. The gel solutions were prepared according to Dewald *et al*.^49^. A 7% running gel was cast upto a height of 3 cms and was overlayed with a 3% stacking gel solution upto a height of 1.5 cms. The affinity chromatography peak eluate was concentrated six fold and 1.5 ml of this concentrated extract (containing about 3 IU AChE) was loaded onto the preparative PAGE. Electrophoresis was carried out at constant current of 15 mA (260 V) for 1 h followed by an 18 h run at 25 mA (330 V). Temperature was maintained at 4 °C throughout the run. A thin longitudinal section of the gel was stained to visualize AChE using the method of Karnosky & Roots^50^. The matching positions of the two AChE bands were cut out from the remaining gel portion and eluted by electroelution.

#### Electroelution

The AChE bands cut out of the preparative gel were macerated in chilled upper tank buffer. The bottom of the glass tube used for the preparative PAGE was sealed with parafilm and a 7% polyacrylamide gel button polymerized. The macerated gel was then transferred to the tube which was then filled to the brim with upper tank buffer. The top of the tube was sealed with a dialysis membrane kept in place with the help of a rubber washer. The tube was inserted into the gel apparatus and the run carried out keeping the anode at the bottom.

Electroelution was carried out for 15 h at a constant current of 36 mA (280 V). On completion, the dialysis membrane was puctured and the eluted AChE withdrawn using a syringe.

### Enzyme kinetics

K_m_ and V_max_ were determined *in vitro* at temperatures from 10 °C to 40 °C at 5 °C intervals, in the substrate concentration range, 0 mM to 1.2 mM ATChI. The constants were determined by the direct linear plot^55^ using a computer programme developed by Crabbe^56^.

#### Molecular weight estimation

The molecular weights of the two purified AChE species were determined by the method of Hendrick and Davis^48,57^ as modified by Bryan^58^: a Ferguson plot using non-denaturing PAGE. The molecular weights of the sub-units were determined by SDS- PAGE^59^.

### Thermal stability and energy of activation

The thermal stability of the two AChE molecular forms purified were tested by heating aliquots of the enzymes in 50 mM phosphate buffer (pH 8.0) in a water-bath for varying intervals of time at different temperatures ranging from 35 °C to 50 °C. Assay was carried out at optimum temperature. The energy of activation for each AChE was determined at *in vitro* temperatures between 10 °C and 40 °C using the Arrhenius plot.

## Data availability

No datasets were generated or analyzed during the current study.
